# Difference in overall and age-specific prevalence of high-risk human papillomavirus infection in Italy: evidence from NTCC trial

**DOI:** 10.1186/1471-2334-13-238

**Published:** 2013-05-24

**Authors:** Iacopo Baussano, Silvia Franceschi, Anna Gillio-Tos, Francesca Carozzi, Massimo Confortini, Paolo Dalla Palma, Margherita De Lillo, Annarosa Del Mistro, Laura De Marco, Carlo Naldoni, Paola Pierotti, Patrizia Schincaglia, Nereo Segnan, Manuel Zorzi, Paolo Giorgi-Rossi, Guglielmo Ronco

**Affiliations:** 1 International Agency for Research on Cancer, Lyon, France; 2 University of Turin, Turin, Italy; 3 ISPO, Florence, Italy; 4 S.Chiara Hospital, Trento, Italy; 5 Imola Hospital, Imola, Italy; 6 Istituto Oncologico Veneto, Padua, Italy; 7 Emilia-Romagna Region, Bologna, Italy; 8 Maggiore Hospital, Bologna, Italy; 9 Centro di Prevenzione Oncologica, AUSL Ravenna, Italy; 10 Centro per la Prevenzione Oncologica (CPO), Turin, Piedmont, Italy; 11 ASP Lazio, Rome, Italy

**Keywords:** Human papillomavirus, Prevalence, Age-specific

## Abstract

**Background:**

Although among women a decreasing prevalence of human papillomavirus (HPV) infection with increasing age has been consistently observed in high-resource countries, different age profiles have been reported elsewhere.

**Methods:**

We compared the age profile of high-risk (HR)-HPV prevalence in nine different areas of Northern and Central Italy by studying the women recruited in the intervention arm of the New Technologies in Cervical Cancer study and tested by Hybrid Capture 2. Differences in the age-distribution of HPV infection were investigated in each centre by the joinpoint approach in a logistic model. 46,900 women aged 25 to 60 years were included in the analysis.

**Results:**

The HR-HPV age-standardised (on Italian population) prevalence ranged from 5.7% (Trento) to 10.3% (Ravenna). HR-HPV prevalence decreased as a logistic function of increasing age in 6 of 9 centres (Trento, Verona, Florence, Bologna, Imola, and Viterbo). The effect of age on HR-HPV prevalence slopes did not differ significantly among these 6 centres, whereas significant heterogeneity in intercepts (p < 0.001) was found, reflecting different overall HR-HPV prevalence between centres. One significant joinpoint was observed in 2 centres (Padua and Ravenna), indicating that the decrease in HR-HPV prevalence by age was better described using a function composed with two logistic segments. In Padua HR-HPV prevalence decreased only slightly up to 39 years but showed a steep downturn thereafter. In Ravenna HR-HPV prevalence decreased steeply down to 45 years of age and then showed a plateau. Finally, in Turin two significant joinpoints were observed: prevalence decreased only after age 29 and showed a plateau after age 39.

**Conclusions:**

Our results showed substantial differences in overall and age-specific HR-HPV prevalence across Italian areas. These findings may be related to different timing of changes in sexual behaviours across regions. Age-specific HR-HPV prevalence in Italy does not support an influence of age *per se*.

## Background

The prevalence of cervical human papillomavirus (HPV) infection varies across populations and according to a woman’s age. Studies from Europe [1-4] and North America [5-7] showed high HPV prevalence in young women with subsequent marked declines after age 25 or so. This pattern was also observed in industrialized countries in South America and Asia, e.g., Argentina [8] and Korea [9]. Some studies in Central [10,11] and South America [12-14] showed a more modest second peak in HPV prevalence in women 45 or 55 years or older. In addition, a few studies found that in some low-resource populations HPV prevalence remains high at any age [15-19].

The very steep increase in the cumulative incidence of HPV infection observed among women who had recently become sexually active suggested that a majority of women acquire the infection in the first few years after sexual debut [6,20]. However, clear evidence emerged from cohort studies suggesting that middle-aged women do not only harbour long-term HPV persistent infections but also new incident infections [21,22]. The geographic heterogeneity in age-specific HR-HPV prevalence in different populations suggests that population-specific factors, including sexual behaviour at different ages and birth cohort, need to be taken into account along with the natural history to characterize the HR-HPV infection epidemiology [23].

Previous studies were, however, based on samples of a few hundreds or few thousands women overall, limiting the possibility of studying age effect in detail. Therefore, we aimed at assessing HR-HPV prevalence and possible variations in the age-distribution of HPV prevalence in 46,900 women from a large multicentre randomised controlled trial conducted across nine different areas in Italy.

## Methods

**Figure 1:**
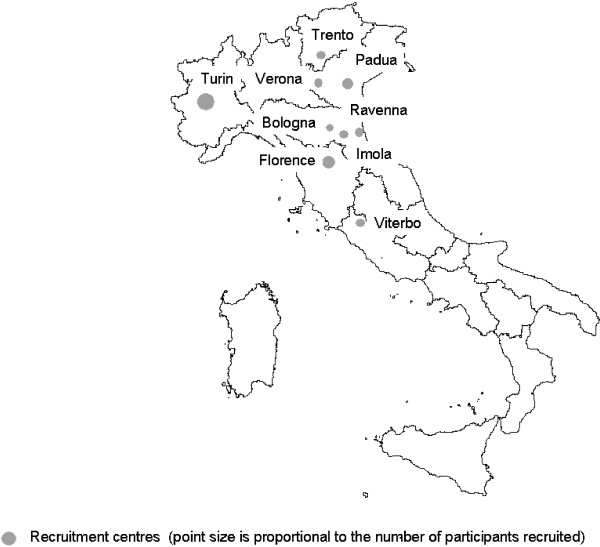
Location of new technologies in cervical cancer study recruitment centres within Italy.

The New Technologies in Cervical Cancer (NTCC) study is a randomized trial of screening methods (conventional cytology *versus* HPV testing) for which the main endpoint was histologic detection of cervical intraepithelial neoplasia of grade 2 or more. Our report is based only on the HPV testing arm of NTCC. NTCC was conducted within organised cervical cancer screening programmes in nine centres in Northern and Central Italy (Turin, Trento, Padua, Verona, Florence, Bologna, Imola, Ravenna and Viterbo) and was considered in determining HPV prevalence. Figure 1 shows the location of participating centres within Italy. Turin is a large industrial city of about 1 million inhabitants. Padua, Bologna and Florence are middle-sized towns of about 2–300,000 inhabitants. The Verona screening programme targeted a mainly rural area close to the town of Verona. The remaining screening programmes included the populations of small towns (50–100,000 inhabitants) and surrounding rural areas. In particular, the Trento screening programme served a mountain area while Ravenna is close to an important touristic resort area on the Adriatic Sea.

Methods of recruitment and randomisation of NTCC study have been previously described [24-26]. Briefly, following an invitation sent to the entire target population, women aged 25–60 years attending a new round of cervical screening were assigned randomly to either conventional cytology or HPV testing arm. Women who were pregnant, reported to be virgin, had undergone hysterectomy, or had been treated for cervical intraepithelial neoplasia (CIN) or cancer in the past 5 years were excluded. Seventy-four percent of eligible women consented to the study and were randomized [27]. All participants provided written informed consent. The study was approved by the ethics committee of the coordinating centre in Turin (Commissione sperimentazioni cliniche della Regione Piemonte - Comitato etico di riferimento regionale, art.7, DM 18 marzo 1998) and by local ethics committees of each participating centre. Baseline characteristics of NTCC participants have been previously described [24-27].

In the HPV testing arm, exfoliated cervical cells were collected using a plastic Ayre’s spatula and a cytobrush, and eluted into the PreservCyt buffer (NTCC phase 1) or in the standard transport medium (Qiagen Incorporated) (NTCC phase 2). The Hybrid Capture 2 (HC2) hybridization assay (Qiagen, Hilden, Germany) targeting HR-HPV types 16, 18, 31, 33, 35, 39, 45, 51, 52, 56, 58, 59 and 68, was used to determine HR-HPV presence with a cut point of 1 relative light unit (RLU) for HPV positivity, as recommended by the manufacturer. High reproducibility of HPV testing between laboratories was documented [28].

HR-HPV status from the first valid HC2 test was considered. For each centre, HR-HPV prevalence was standardized to the 2004 Italian female population in the same age groups for year 2004 aged 25–60 years [29]. The heterogeneity in age-standardized HPV prevalence across centres was assessed using the inverse-variance method and tested with the Q statistics from the random effects pooling method proposed by DerSimonian and Laird [30].

The age-distribution of HR-HPV prevalence in each recruitment centre was assessed performing a logistic joinpoint regression analysis. This method allows characterizing the age-specific prevalence using joint linear segments with different slopes, where the joinpoints represent the points of change in slope. If no significant joinpoints were identified, we characterized the age-specific HR-HPV prevalence profiles using a standard logistic regression with studied differences between centres and logistic effect of age as suggested by the intercepts and the slopes. For the centres without any significant joinpoint we tested the significance (significance level < 0.05) of differences between centres in intercepts, reflecting different overall HR-HPV prevalence, and age slopes, reflecting different declines of HR-HPV prevalence as a function of age. To test for significant differences in intercepts between centres we performed a log-likelihood ratio test comparing two logistic regression models: one accounting only for age as independent variable, the other for both age and centres. Similarly, to test for different declines of HR-HPV prevalence as a function of age among centres, we tested the statistical significance of the interaction between centres and age performing a log-likelihood ratio test.

To test for the presence of statistically significant (significance level < 0.05) joinpoints and to estimate their value, we used the package “ljr” from the CRAN repository [31]. In particular, the used function tests, based on the likelihood ratio test statistic, the null hypothesis of *j* joinpoint(s) *versus* the alternative of *j + 1* joinpoint(s). The p-value is determined by a Monte Carlo method [32]. We allowed for up to three possible joinpoints (*j*_*max*_ = 3) and set as significance level < 0.05). All the other statistical analyses were performed using STATA software (StataCorp. 2011. Stata Statistical Software: Release 12. College Station, TX: StataCorp LP).

## Results

A total of 47,369 women were randomly assigned to the HPV testing arm. After the exclusion of 469 women who did not have any valid HPV test, 46,900 women could be included in the present analysis.

**Figure 2:**
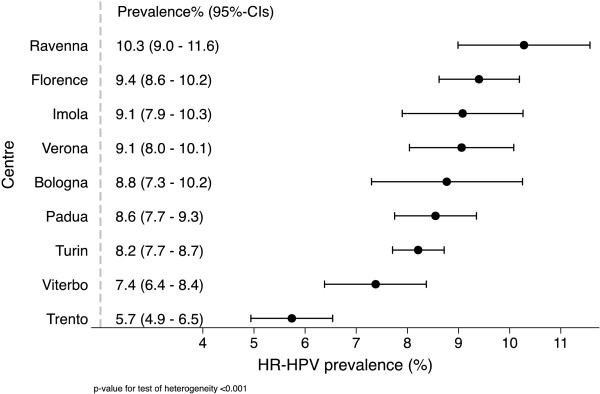
**The age-standardised prevalence, by recruitment centre.** CI: confidence interval; HR: high-risk; HPV: human papillomavirus.

Table 1 shows the number of women found to be positive for HR-HPV types by centre and age group. The overall HR-HPV unadjusted prevalence was 8.4%; the overall age-specific prevalence steadily decreased as a function of age, from 15.1% at age 24–29 to 4.0% at age 55–60. Additional file 1: Figure S1 section, shows the HR-HPV prevalence (%) as a function of age for each recruitment centre. Age-standardized prevalence varied significantly, as assessed using the inverse-variance method and tested with the Q statistics (p-value <0.001), by centre with the lowest values in Trento (5.7%; 95% confidence interval (CI): 4.9-6.5) and Viterbo (7.4%; 95% CI: 6.4-8.4) and the highest value in Ravenna (10.3%; 95% CI: 9.0-11.6) (Figure 2). All the remaining centres had values between 8.2% and 9.4%.

**Table 1:** Prevalence of hybrid capture 2 positive-women by age and recruitment centre

**HPV+/Tested women (prevalence%)**								
**Age groups**	**25-29**	**30-34**	**35-39**	**40-44**	**45-49**	**50-54**	**55-60**	**Total**
Verona	69/587 (11.8%)	104/736 (14.1%)	62/632 (9.8%)	43/533 (8.1%)	28/322 (8.7%)	37/476 (7.8%)	20/449 (4.5%)	363/3735 (9.7%)
Padua	142/994 (14.3%)	124/1013 (12.2%)	134/948 (14.1%)	59/799 (7.4%)	33/551 (6.0%)	10/262 (3.8%)	13/723 (1.8%)	515/5290 (9.7%)
Ravenna	65/340 (19.1%)	67/521 (12.9%)	68/591 (11.5%)	43/628 (6.8%)	36/582 (6.2%)	31/548 (5.7%)	38/541 (7.0%)	348/3751 (9.3%)
Florence	150/926 (16.2%)	141/1045 (13.5%)	138/1277 (10.8%)	103/1306 (7.9%)	90/1227 (7.3%)	58/1204 (4.8%)	48/1129 (4.3%)	728/8114 (9.0%)
Imola	58/325 (17.8%)	50/415 (12.0%)	48/528 (9.1%)	41/510 (8.0%)	28/373 (7.5%)	17/406 (4.2%)	17/357 (4.8%)	259/2914 (8.9%)
Turin	274/1761 (15.6%)	252/1909 (13.2%)	166/2219 (7.5%)	132/2142 (6.2%)	99/1860 (5.3%)	99/1904 (5.2%)	84/2015 (4.2%)	1106/13810 (8.0%)
Bologna	28/137 (20.4%)	29/262 (11.1%)	43/418 (10.3%)	35/480 (7.3%)	24/413 (5.8%)	22/432 (5.1%)	16/428 (3.7%)	197/2570 (7.7%)
Viterbo	71/507 (14.0%)	35/390 (9.0%)	36/501 (7.2%)	27/460 (5.9%)	34/440 (7.7%)	17/376 (4.5%)	14/415 (3.4%)	234/3089 (7.6%)
Trento	43/400 (10.8%)	41/572 (7.2%)	39/634 (6.2%)	34/632 (5.4%)	22/501 (4.4%)	16/459 (3.5%)	12/429 (2.8%)	207/3627 (5.7%)
Total	900/5977 (15.1%)	843/6863 (12.3%)	734/7748 (9.5%)	517/7490 (6.9%)	394/6269 (6.3%)	307/6067 (5.1%)	262/6486 (4.0%)	3957/46900 (8.4%)

**Figure 3:**
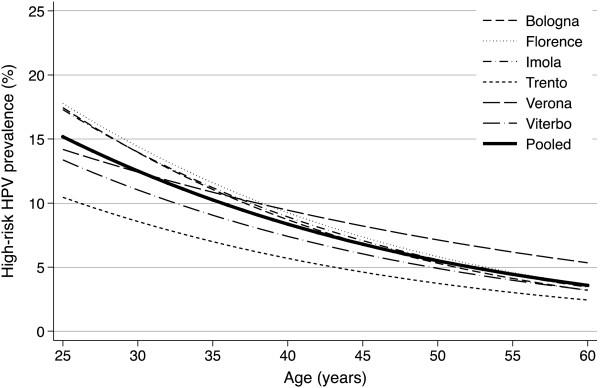
**Model-based age-specific prevalence for centres without significant joinpoint (i.e. (Verona, Trento, Bologna, Imola, Florence and Viterbo).** The thick solid curve shows the age-specific prevalence pooled across recruitment centres.

No significant joinpoints were observed in six of the nine centres (Verona, Trento, Bologna, Imola, Florence and Viterbo). HR-HPV prevalence in these centres decreased as a logistic function of increasing age. Significant heterogeneity in intercepts (p < 0.001), as assessed performing a log-likelihood ratio test, was found between centres, reflecting different overall HR-HPV prevalence (Figure 3). While HR-HPV prevalence showed a steeper decline with age in Bologna, Florence and Imola than in Trento, Verona and Viterbo, the test for heterogeneity of age slopes did not reach statistical significance (p = 0.08).

**Figure 4:**
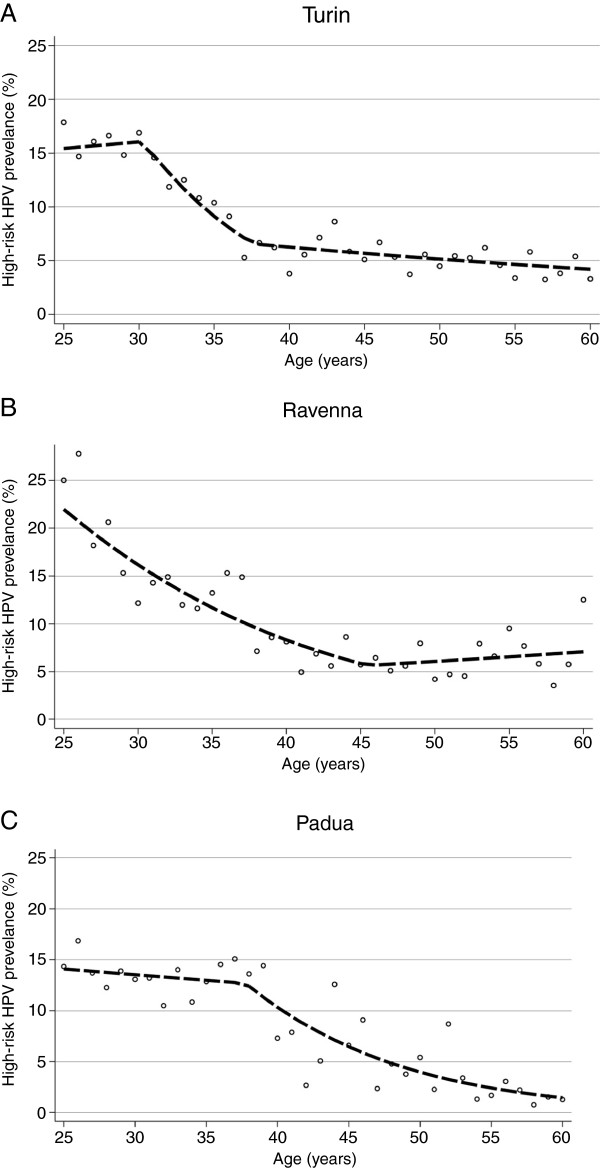
**Age-specific prevalence and the fitted regression according to the joinpoint logistic regression analysis for centres with significant joinpoint (i.e. Turin –Panel A, Ravenna – Panel B, and Padua – Panel C).** HC2: Hybrid Capture 2.

Figure 4 shows age-specific prevalence and the joinpoints regression for the three centres where at least one statistically significant (p < 0.05) joinpoint was found. In Turin two significant joinpoints were observed: HR-HPV prevalence remained high up to age 30 years, decreased steeply between age 30 and 38 and maintained a very slow decline after age 38. In Ravenna HR-HPV prevalence decreased until 45 years of age and reached a plateau thereafter, with a slightly higher prevalence observed in the oldest women. In Padua, HR-HPV prevalence showed a very slow decline between age 25 and 39, with a higher prevalence for women aged 35–39 than in any other centre (14.1% vs. 9.5% on average), and subsequently had a steep downturn with the lowest prevalence observed in the oldest age groups.

## Discussion

We observed geographical variation in age-standardized and age-specific prevalence of HR- HPV infection in the Italian centres participating in the NTCC study; with the highest age-standardized prevalence in Ravenna (close to a touristic sea resort area) almost twice that of Trento (a mainly rural mountain area), the lowest prevalence. Given that relevant differences in biological features of HR-HPV infections across Italian regions are implausible, the observed geographical variations are likely to reflect differences in sexual behaviour across Italian areas.

In addition, although in all study centres HR-HPV prevalence followed the general pattern of decrease with increasing age typical of industrialised countries, qualitative differences were found (Ravenna, Turin and Padua) in the shape of age specific prevalence. In the majority of NTCC centres HR-HPV prevalence decreased as a logistic function of increasing age, i.e., a logistic function described HR-HPV prevalence reduction within the considered 25-to-60-year-old age range. In Turin and Padua, however, women showed higher HR-HPV infection prevalence up to about age 30 and 40 years, respectively. The same age-distribution of HR-HPV prevalence in Turin had been found in a previous smaller study [4]. Consistent high prevalence of HR-HPV infection in 25-to-30 and 25-to-39-year-old women in Turin and Padua, respectively, the two largest Northern Italian towns included in the NTCC study, could reflect the increasing delay in age at first marriage [33]. In turn, this may result in more sexual promiscuity before marriage, and thus increased HPV exposures. Also, high prevalence of HR-HPV infection in Turin and Padua could derive from birth cohort effects mainly due to sexual behavioural changes in younger birth cohorts, leading to earlier initiation of sexual activity compared to elsewhere in Italy. A similar delay in the decline in HR-HPV prevalence was reported in Poland [34]. Ravenna was the centre that had the highest overall HR-HPV prevalence with a second peak in prevalence detected above age 45, possibly reflecting substantial HR-HPV exposures among middle-aged women or their partners in an area where tourism has been flourishing for at least five decades.

Of notice, a steeper decrease in HR-HPV prevalence with age than that found in the NTCC study was observed in a study conducted in Central and Southern Italy [35]. This finding suggests that age-specific sexual patterns across Italy differ, with a more pronounced reduction of sexual activity or promiscuity with aging in Southern Italy. Geographical differences in sexually related behaviours across Italy have also been reported from a nation-wide survey on sexual activities [36] and contraceptive use [37]. Unfortunately, surveys of sexual behaviour in Italy are sparse and do not allow clear comparisons between birth cohorts.

Differences in sexual habits and birth cohort effects may herald different future changes in the incidence of precancerous and cancerous lesions of the cervix in different parts of Italy. Increases in both incidence [38] of and mortality [39] from cervical cancer in younger cohorts have been observed in many European countries and in others, like England, were plausibly masked by screening efficiency [40].

The variation in the shape of age-specific HR-HPV prevalence observed between centres in the NTCC study could also derive from different population mixing. On account of important migratory waves, for instance, less than half of the population living in Turin in 2011 was born in the city. As for many other large towns across industrialized countries, Turin is constantly subject to rural-to-urban migrations. Main reasons for immigration from both other Italian areas and abroad are studying and working purposes. We speculate that the constant inflow of relatively young immigrants may have an impact on sexual promiscuity and transmission chain of HPV infections. The consequences of immigration on older birth-cohorts are of less straightforward interpretation. Demographically relevant migrations to Turin, initially from Southern and North-eastern Italy, started in the 1960s. Almost half the women migrated to Turin from Southern Italy arrived during such period and would have not fallen in the age range eligible for the NTCC study [41].

Our study has strengths and weaknesses. Study population is large and can be considered well representative of women aged between 25 and 60 years who participate in screening in the study areas. In a previous study of the determinants of screening attendance in Turin [42], we found no significant effect of age, education, marital status or place of birth on response to invitation to screening. Given the standardization and quality control assurance of organized screening programmes across Italy, any selection bias is unlikely to have applied differently to different study centres or age groups thus reassuring on the validity of the present comparisons. The use of a well-validated HPV testing method is another asset of the present study. A few weaknesses should also be mentioned. Despite the large size of the present study, the power to detect significant joinpoints in age-specific HR-HPV prevalence was limited in the smaller centres. Lack of inclusion of certain geographic areas, in particular from southern Italy, makes our current estimate of HR-HPV prevalence not equivalent to a national average. Another weakness is lack of individual information on place of birth, marital history, and sexual habits that could have helped elucidating some of the determinants of variations in HR-HPV prevalence.

## Conclusions

In conclusion, we observed substantial differences in overall and age-specific HPV prevalence across Italian areas. These findings may be related to differences in sexual behaviour of women in different age groups and birth cohorts and in population mixing across study areas. Awareness of the existence of age or cohort effects may be useful in predicting trends in precancerous and cancerous lesions of the cervix and modelling the future impact of screening or HR-HPV vaccination in different Italian areas. In any case, the heterogeneity we observed does not support an influence of age *per se* in HR-HPV prevalence.

## Abbreviations

CI: Confidence interval; CIN: Cervical intraepithelial neoplasia; HR: High-risk; HPV: Human papillomavirus; HC2: Hybrid capture 2; NTCC: New technologies in cervical cancer; RLU: Relative light unit.

## Competing interests

The authors declare that they have no competing interests.

## Authors’ contributions

SF, GR, IB conceived the study. GR, SF, PGR and NS were involved in the study design. AGT, FC, MC, PDP, MDL, ADM, LDM and CN collected data. PP, PS, NS, MZ and PGR were responsible for quality control of data and algorithms. IB, GR performed statistical analysis and data analysis and interpretation with SF. IB, GR and SF drafted the manuscript and NS, FC and PGR edited it. All authors read and approved the final manuscript.

## Pre-publication history

The pre-publication history for this paper can be accessed here:

http://www.biomedcentral.com/1471-2334/13/238/prepub

## Supplementary Material

Additional file 1: Figure S1 Supplementary information. Click here for file
