# Supplementary material for: Difference in overall and age-specific prevalence of high-risk human papillomavirus infection in Italy: evidence from NTCC trial
Source: BMC Infect Dis. 2013 May 24;13:238. doi: 10.1186/1471-2334-13-238 (PMC3669053; doi:10.1186/1471-2334-13-238)
Supplement: Additional file 1: Figure S1 — Supplementary information. [file 1471-2334-13-238-S1.docx]

**Supplementary Information**

**Difference in overall and age-specific prevalence of high-risk human papillomavirus infection in Italy: evidence from NTCC trial**

Iacopo Baussano^1*^, Silvia Franceschi^1^, Anna Gillio-Tos^2^, Francesca Carozzi^3^, Massimo Confortini^3^, Paolo Dalla Palma^4^, Margherita De Lillo^5^, Annarosa Del Mistro^6^, Laura De Marco^2^, Carlo Naldoni^7^, Paola Pierotti^8^, Patrizia Schincaglia^9^, Nereo Segnan^10^, Manuel Zorzi^6^, Paolo Giorgi-Rossi^11^, Guglielmo Ronco^10^

^1^International Agency for Research on Cancer, Lyon, France; ^2^University of Turin, Turin, Italy; ^3^ISPO, Florence, Italy; ^4^S.Chiara Hospital, Trento, Italy; ^5^Imola Hospital, Imola, Italy; ^6^Istituto Oncologico Veneto, Padua, Italy; ^7^Emilia-Romagna Region, Bologna, Italy; ^8^Maggiore Hospital, Bologna, Italy; ^9^Centro di Prevenzione Oncologica, AUSL Ravenna, Italy; ^10^Centro per la Prevenzione Oncologica (CPO). Turin, Piedmont Italy; ^11^ASP Lazio, Rome, Italy.

Figure S1. HR-HPV prevalence (%), by age and recruitment centre.
